# Cross-Resistance of the Codling Moth against Different Isolates of Cydia pomonella Granulovirus Is Caused by Two Different but Genetically Linked Resistance Mechanisms

**DOI:** 10.3390/v13101952

**Published:** 2021-09-29

**Authors:** Annette J. Sauer, Eva Fritsch, Karin Undorf-Spahn, Kento Iwata, Regina G. Kleespies, Madoka Nakai, Johannes A. Jehle

**Affiliations:** 1 Julius Kühn-Institut—Federal Research Centre for Cultivated Plants, Institute for Biological Control, 64287 Darmstadt, Germany; annette.sauer@gmx.de (A.J.S.); eva.fritsch@julius-kuehn.de (E.F.); karin.undorf-spahn@julius-kuehn.de (K.U.-S.); regina.kleespies@julius-kuehn.de (R.G.K.); 2 Department of Applied Biological Science, Faculty of Agriculture, Tokyo University of Agriculture and Technology, Tokyo 183-8509, Japan; ssc011308@gmail.com (K.I.); madoka@cc.tuat.ac.jp (M.N.)

**Keywords:** virus resistance, insects, *Cydia pomonella*, baculovirus, midgut, bioassay

## Abstract

Cydia pomonella granulovirus (CpGV) is a widely used biological control agent of the codling moth. Recently, however, the codling moth has developed different types of field resistance against CpGV isolates. Whereas type I resistance is Z chromosomal inherited and targeted at the viral gene *pe38* of isolate CpGV-M, type II resistance is autosomal inherited and targeted against isolates CpGV-M and CpGV-S. Here, we report that mixtures of CpGV-M and CpGV-S fail to break type II resistance and is expressed at all larval stages. Budded virus (BV) injection experiments circumventing initial midgut infection provided evidence that resistance against CpGV-S is midgut-related, though fluorescence dequenching assay using rhodamine-18 labeled occlusion derived viruses (ODV) could not fully elucidate whether the receptor binding or an intracellular midgut factor is involved. From our peroral and intra-hemocoel infection experiments, we conclude that two different (but genetically linked) resistance mechanisms are responsible for type II resistance in the codling moth: resistance against CpGV-M is systemic whereas a second and/or additional resistance mechanism against CpGV-S is located in the midgut of CpR5M larvae.

## 1. Introduction

Baculoviruses form a large group of dsDNA viruses, which are specific for the larval stages of insects of the orders Lepidoptera, Hymenoptera, and Diptera [1]. Because of their narrow host ranges and high virulence to early insect instars, they are widely used as commercial biocontrol agents replacing the use of environmentally detrimental chemical pesticides [2,3]. The Cydia pomonella granulovirus (CpGV) (belonging to the genus *Betabaculovirus*) is registered in most pome fruit growing countries worldwide and is widely applied as bio-control agent in integrated and organic apple, pear, and walnut production to control codling moth (*Cydia pomonella*) caterpillars. CpGV was first discovered in Mexico, but further natural geographic isolates were found in the Caucasus area, Europe, North and South America, South Africa, and recently in China [4,5,6,7,8,9,10,11,12]. The genome of CpGV is 120.8–124.3 kbp in size and encodes between 137 and 142 open reading frames (ORFs) depending on the isolate [13,14]. CpGV isolates can be classified into seven genome groups A–G [11,14,15], representing different phylogenetic lineages.

The CpGV infection pathway in the insect larvae is initiated by an oral uptake of viral occlusion bodies (OB), containing a single viral nucleocapsid. After OB have dissolved in the alkaline environment of the larval midgut releasing occlusion-derived viruses (ODV), infection of midgut epithelial cells is initiated [16]. After the initial infection of midgut epithelial cells, a second phenotype, termed budded virus (BV), is produced and eventually released through the basal midgut membrane. The BV is responsible for the systemic spread of the viral infection to other larval tissues, presumably similar as for nucleopolyhedroviruses [17,18]. At the end of the infection process, new virus OB are produced and eventually released from the disintegrating larval cadavers [16].

The Mexican isolate CpGV-M has been used for several decades in commercial CpGV products in Europe and most other countries worldwide [19,20], until the first cases of field resistance of the codling moth against CpGV-M were discovered in 2005 [21,22]. More than 40 apple orchards with codling moth populations resistant to CpGV-M have been identified since then in Austria, the Czech Republic, Germany, France, Italy, the Netherlands, and Switzerland [23,24,25]. Because most of these resistant codling moth populations could be successfully controlled by newly registered resistance-breaking CpGV isolates, it was assumed that resistance to CpGV follows a widely spread, common mechanism, called type I resistance [26]. It has been shown that type I resistance is inherited by a single, dominant allele, located on the Z chromosome [23,24,27]. Type I resistance is targeted only against CpGV isolates from genome group A, such as CpGV-M, whereas CpGV isolates from other genome groups were still virulent in resistant CM populations [10,23,24,28,29,30]. By studying the laboratory-selected codling moth strain CpRR1, the viral gene *pe38* of CpGV-M was proposed as the main target of type I resistance, because replacement of this gene in CpGV-M by *pe38* from the resistance-breaking isolate CpGV-S enabled the recombinant CpGV-M also to overcome resistance [14]. Type I resistance is further characterized by a systemic and early block of CpGV replication, which occurs in all larval instars [28,31].

Recently, a further type of CpGV resistance was reported from apple orchards in Germany. This type II resistance is also dominant but autosomally inherited [26]. Type II resistance appeared to be targeted at CpGV isolates from genome groups A, C, D, E, and G, whereas the isolates CpGV-E2 (genome group B) and CpGV-ZY2 and -JQ (genome group F) were able to overcome this resistance [10,11,26,32]. Selection of this field population for five generations on either CpGV-M (genome group A) or CpGV-S (genome group E) had rendered two codling moth strains, namely CpR5M and CpR5S, which were cross-resistant to both genome groups, suggesting a different resistance mechanism as found for type I resistance [32]. In addition to type II resistance, further forms of CpGV resistance, not following the CpGV isolate-dependent inheritance or susceptibility patterns of type I or type II resistance, were recently identified in Germany, Italy, and France [33,34].

To further elucidate the resistance mechanism in the codling moth larvae with type II resistance, we conducted comparative in vivo experiments using the isolates CpGV-M and -S. Whereas CpGV-M was not able to infect type II resistant codling moth larvae, neither by OB ingestion nor by BV injection, CpGV-S was able to overcome resistance when injected directly in the hemocoel. Our experiments revealed clear evidence for two independent but co-inherited resistance mechanisms.

## 2. Materials and Methods

### 2.1. Viruses and Insects

Different wild type isolates of Cydia pomonella granulovirus (CpGV) were used: isolate CpGV-M (genome group A) [4], isolate CpGV-S (group E) [14]. All OB were stored at −20 °C until used. Quantification of virus stocks was performed by OB counting with a light microscope (Leica DMRBE; Leitz, Wetzlar, Germany) in dark-field optics with the Petroff-Hausser counting chamber (depth 0.02 mm) (Hausser Scientific, Horsham, PA, USA).

The codling moth strain CpS originated from a colony fully susceptible to CpGV [27]. The codling moth strain CpR5M originated from the field population NRW-WE, which was selected on CpGV-M for five generations; CpR5M showed cross-resistance against both CpGV-M and CpGV-S [32]. The different codling moth strains were reared in the laboratory at 26 °C with 16/8 h light/dark photoperiod and 60% relative humidity; larvae were kept on a semi-artificial, modified diet of Ivaldi-Sender [35].

### 2.2. Resistance Testing

Neonates of CpS or CpR5M were tested for resistance as described before [36]. In brief, the applied discriminating concentration of 5.8 × 10^4^ OB/mL of diet causes >95% mortality in CpS neonates (L1) in bioassays after seven days [27]. Mortality of larvae was determined at 1, 7, and 14 days post inoculation; only larvae surviving 1-day post inoculation were introduced to the test. For co-infection tests, codling moth larvae were exposed to different ratios of CpGV-M:CpGV-S (90:10, 50:50 and 10:90), also at a final concentration of a 5.8 × 10^4^ OB/mL diet. Treatment mortality was corrected for control mortality [37]. At least 30 larvae were used in each of the assays, which were independently repeated at least three times with CpR5M and one to three times with CpS.

### 2.3. Instar-Specific Assays

First to fifth instars (L1 to L5) were exposed to OB of CpGV-M and -S at a concentration of 2.0 × 10^5^ OB/mL incorporated into the diet. This concentration caused >95% mortality in all larval (L1 to L5) stages of CpS after seven days [27]. At least 20–35 larvae of CpR5M or CpS were used in each assay and the virus-induced mortality was assessed daily for 14 days; all mortality data were corrected with control mortality [37]. Three independent replicates were performed.

### 2.4. Budded Virus Preparation

Budded virus (BV) of CpGV isolates was produced as described in [33], with some modifications. Fourth instars (L4) of the susceptible strain CpS were orally infected with 1 × 10^4^ OB of CpGV-M or CpGV-S, which were pipetted on a small piece of diet. Larvae that ingested the whole piece of diet within 12 h were transferred to a virus-free diet for three days. Hemolymph was collected by cutting off the second proleg of 20 anesthetized larvae and pooled in 200 µL IZD04 cell culture medium containing a small crystal of N-Phenylthiourea (Sigma-Aldrich, St. Louis, MO, USA). Hemolymph of uninfected L4 larvae of CpS was included as a negative control. After centrifugation at 1000× *g* at 4 °C for 5 min, the supernatant containing the BV was stored at 4 °C for a maximum of one month.

### 2.5. Quantitative PCR

BV concentration of CpGV-M and CpGV-S was estimated by quantitative PCR (qPCR), using an internal OB standard of three-fold dilutions between 7.5 × 10^4^ and 7.5 × 10^8^ OB/mL [36]. OB standard suspensions were dissolved in 100 mM Na_2_CO_3_ at 37 °C for 30 min. Moreover, 100 µL each of OB standard suspensions and BV hemolymph were purified by Ron’s Tissue DNA Mini Kit^®^ (BIORON GmbH, Ludwigshafen, Germany). DNA was eluted in 100 µL elution buffer (EB) provided with the kit and used as a template for qPCR. The qPCR reaction was performed according to the protocol of Maxima SYBR Green qPCR^®^ (Thermo Fisher Scientific, Waltham, MA, USA). Briefly, each PCR sample consisted of 2 µL of standard or sample DNA template mixed with 1 µL 0.2 pM of each of the granulin gene specific oligonucleotides nested_PRCP1_upper (5′-GGC CCG GCA AGA ATG TAA GAA TCA-3′) and nested_PRCP1_lower (5′-GTA GGG CCA CAG CAC ATC GTC AAA-3′) [33], 12.5 µL 1 × Maxima SYBR Green qPCR^®^ Master Mix, and 8.5 µL bidistilled H_2_O, resulting in a total reaction volume of 25 µL. Negative control contained 2 µL of bidistilled H_2_O instead of DNA template. All qPCR reactions were started with a denaturation step of 5 min at 95 °C, followed by 44 cycles of denaturation (95 °C for 30 s), primer annealing (60 °C for 30 s), elongation (72 °C for 30 s), and a final elongation step (72 °C for 7 min). Melting curve analysis was performed from 50 °C to 95 °C with an increment of 0.5 °C each 10 s. The amount of PCR product copies in the BV samples were calculated and extrapolated on the basis of the OB derived DNA standard with the Bio-Rad CFX Manager (3.1) software according to the assumption 1 CpGV OB = 1 CpGV-BV.

### 2.6. Intra-Hemocoelic BV Injections

Five microliters of diluted BV suspension corresponding to a DNA concentration equivalent to that of 1 × 10^6^ OB/mL of either CpGV-M or CpGV-S were injected into the hemocoel of anesthetized L4 larvae of CpS or CpR5M, using a Hamilton syringe (Hamilton, Bonaduz, Switzerland). Hemolymph of uninfected CpS larvae was injected as control. After recovering from the injection, larvae were transferred to virus free diet and virus-induced mortality was recorded 14 days post injection. Larvae, which died within five days because of injection treatment and without virus symptoms, were excluded from the experiment. Bioassays included 10–15 L4 larvae for each virus strain and at least three independent bioassay replicates were performed.

### 2.7. Occlusion-Derived Virus Production and Labeling with R-18

Occlusion-derived virus (ODV) of CpGV-M and CpGV-S were prepared as previously described [38,39], with some modifications. 500 µL of 1 × 10^10^ OB/mL of either CpGV-M or CpGV-S suspension were centrifuged at 20,800× *g* for 10 min. The pellet containing the OB was resuspended and incubated in 450 µL DAS Buffer (alkaline saline, 100 mM Na_2_CO_3_, 100 mM NaCl, pH 11.5) for 30 min at 37 °C to release the ODV from OB. The ODV suspension was neutralized by adding 100 µL 1 M Tris-HCl (pH 6.5) and incubated at room temperature for 20 min. After another centrifugation step at 2060× *g* for 10 min, the ODV concentration in the supernatant was estimated by using the BCA Protein Assay^®^ (Thermo Fisher Scientific, Waltham, MA, USA) and immediately used for labeling. For binding and fusion assays, the ODV were labeled with the self-quenching fluorescent probe octadecyl rhodamine B chloride (R-18) (Thermo Fisher Scientific, Waltham, MA, USA) [40]. Labeled ODV were kept at 4 °C in the dark for a maximum of one month until using.

### 2.8. ODV and Fluorescence Dequenching Assay

For the fluorescence dequenching assays (for details see [39,40]), CpS and CpR5M larvae were reared on virus-free diet until they reached L4. Then, larvae were starved over-night and then orally inoculated with small pieces of diet supplied with 2 µL of labeled ODV at a concentration of 2.4 µg ODV/larvae or with water as a negative control. After the larvae had ingested the piece of diet, they were divided into two cohorts. Because no differences were assessed in binding and fusion efficacy between 30 and 120 min post-infection in ODV assays for previously studied nucleopolyhedroviruses, a time interval of 1 h from inoculating labeled-ODV to dissection of midguts was applied. One cohort of larvae was anesthetized in diethyl ether vapor for 2–3 min and used for dissection of the midgut for the fluorescence dequenching assay. Midgut epithelium of each larva was separated from the basal lamina as previously described [40] and briefly washed in 50 µL separation buffer (100 mM KCl, 100 mM EGTA, 100 mM Na_2_CO_3_, pH 9.5). To confirm the infectivity of the ODV preparation in CpS and CpR5M larvae, the second cohort of infected larvae was transferred to the virus-free diet and incubated at 26 °C with 16/8 h light/dark photoperiod and 60% relative humidity. Dead larvae were recorded seven days post-infection and at least 10 individuals were used for each replicates. Five to six independent replicates were conducted for the ODV bioassay.

Determination of ODV binding was done immediately or samples were kept at −70 °C in the dark until measurement of binding and fusion as described below. At least six larvae were used for each replicate and five to six replicates were undertaken for the fluorescence dequenching assay. Collected midgut epithelial cells suspended in 50 µL of separation buffer were transferred to a 8-tube strip (Bio-Rad, Hercules, CA, USA), and fluorescence was measured for relative fluorescence units (RFUs) for 10 s at 22 °C and 560 nm (excitation) and 610 nm (emission) using a CFX96 Touch^TM^ Real-Time PCR Detection System (Bio-Rad, Hercules, CA, USA). To quantify the total amount of labeled ODV, Triton X-100 (Sigma-Aldrich, St. Louis, MO, USA) with the final concentration of 1% was added to the samples and incubated over night at 4 °C in the dark to allow solubilization of R-18. To determine ODV fusion, RFUs was measured again as described. Fifty µL of labeled ODV of CpGV-M or CpGV-S were measured as a positive control to calculate the total amount of ODV (for details see [41]). Measured RFUs were corrected for background fluorescence associated with the midgut epithelial cells from control larvae fed with water. Four to ten larvae were examined for each replicate and four to five replicates were performed.

### 2.9. Statistical Analysis

Statistical analyses were conducted with ANOVA Scheffé test of the Agricolae Package of RStudio (RStudio edition 2.3.4.4.) (RStudio, PBC, Boston, MA, USA). Box-plot analyses were conducted using RStudio. Prior to statistical analysis, the treatment mortality was corrected for control mortality using Abbott’s formula [37].

## 3. Results

### 3.1. Mortality of CpS and CpR5M Larvae on Different CpGV Isolates

**Figure 1:**
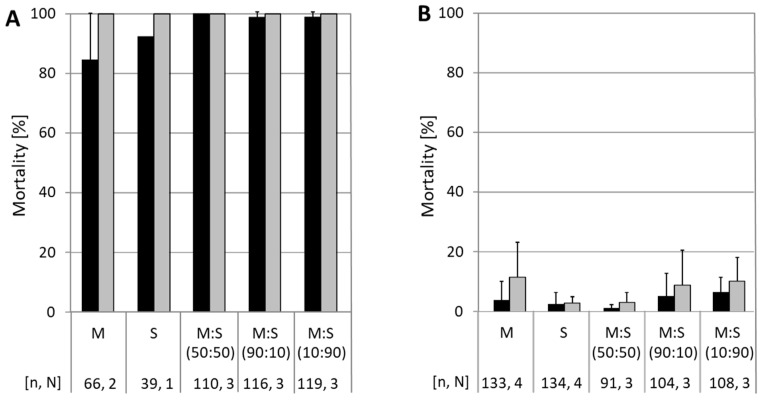
Resistance testing in CpS and CpR5M larvae with CpGV-M (M) and CpGV-S (S) and its combination at different ratios. Mortality of CpS (panel **A**) and CpR5M (panel **B**) neonates tested for resistance on artificial diet mixed with occlusion bodies of CpGV-M or CpGV-S or mixtures of both, all at a final concentration of 5.8 × 10^4^ OB/mL. Mixtures of CpGV-M and CpGV-S were applied at ratios of 50:50, 90:10, or 10:90. Abbott-corrected mean mortality and standard deviations (error bars) were determined at 7 (black bars) and 14 days (gray bars) post-infection. Total number of tested individuals (n) and number of independent replicates (N) are indicated below the charts.

Neonate larvae of the susceptible CpS strain were exposed to a discriminating concentration of 5.8 × 10^4^ OB/mL of either CpGV-M or CpGV-S alone, or of mixtures of CpGV-M:CpGV-S at different ratios. Minimum mortality in all experiments was 84% after 7 days and up to 100% after 14 days, proving the activity of the OB (Figure 1A). When neonates of the resistant codling moth strain CpR5M were tested, mortality was generally below 10% on CpGV-M, CpGV-S, and mixtures of both, even after 14 days of exposure. This finding indicated that neither CpGV-M nor CpGV-S, nor the mixtures were able to break resistance in CpR5M (Figure 1B).

### 3.2. Instar-Specific Assay

**Figure 2:**
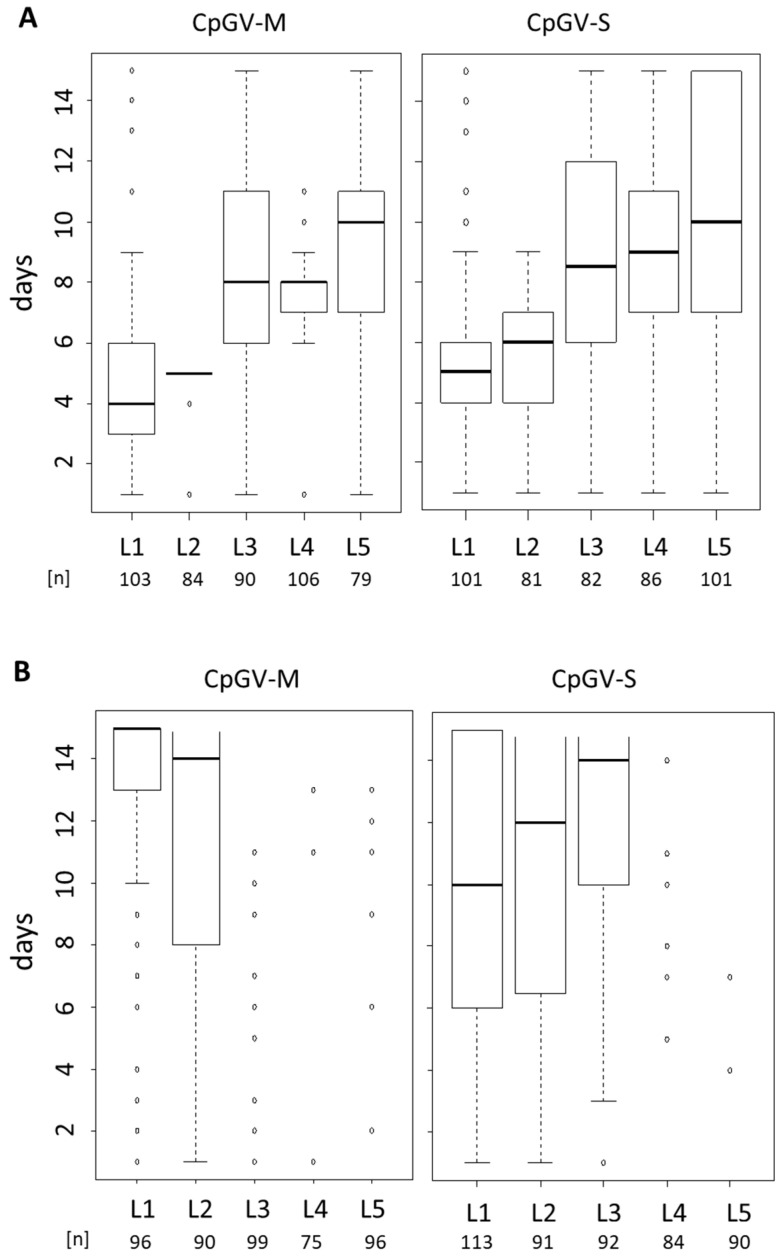
Box-plot analysis of instar-specific (L1–L5) time-to-death of CpS (panel **A**) and CpR5M (panel **B**) larvae subjected to CpGV-M, CpGV-S at a concentration of 2.0 × 10^5^ OB/mL for 14 days. Mortality was recorded daily, open box indicates the 25–75% percentile of time-to-death, bold horizontal lines in the box give the day when 50% of test animals died, vertical dotted lines indicate the days when >0% (lower end, excluding outliers) and 100% (upper end, excluding outliers) mortality were observed, circles stand for outliers. Larval stage (L1–L5) and total number of tested individuals (n) of three independent replicates are given under the box-plot.

To investigate whether resistance to CpGV-M and CpGV-S is related to a particular larval age, different instars (L1–L5) of CpS and CpR5M were infected using a single virus concentration of 2 × 10^5^ OB/mL. The OB concentration normally causes mortality of >95% for all instars of CpS exposed to CpGV-M, CpGV-S, as it is expected for fully susceptible codling moth larvae [27] (Figure 2A). The median time-to-death of CpS in all virus treatments was between 4 days for L1 and 11 days for L5 larvae, and almost all larvae died within the test period.

For CpR5M, a highly reduced susceptibility to both viruses was detectable in all larval stages; most larvae, especially older instars, survived the treatments (Figure 2B). Median time-to-death caused by CpGV-M was only achieved for L1 and L2 larvae at 15 and 14 days post-infection, respectively, whereas for older instars, marginal mortality was observed. CpGV-S showed some activity at least in early instars L1 to L3 with 50% mortality after 10 to 14 days (Figure 2B). These small differences suggested that for CpR5M the efficacy of CpGV-S was slightly higher than that of CpGV-M. In addition, a certain age-dependent increase of median time to death was visible for both strains CpS and CpR5M.

### 3.3. BV Injection Assay

To investigate if resistance of CpR5M against CpGV-M and CpGV-S is systemic or related to oral infection, BV suspensions were injected into the hemocoel of L4 larvae of CpR5M and CpS to bypass the per os infection pathway. The appropriate amount of BV to be applied to the larvae was determined by injecting different concentrations of CpGV-M into L4 larvae of CpS, resulting in mortality between 19.6% and 100% after 14 days (Table 1). Finally, a concentration of 5000 BV/larvae, which caused more than 73% mortality whereas a moderate number of BV was applied (Table 1), was chosen in the following BV injections of CpS and CpR5M larvae. When using this BV concentration, mortality of 73.2% and 94.0% was obtained with CpGV-M and CpGV-S, respectively, in CpS larvae (Table 2). In CpR5M larvae, however, injection with BV of CpGV-M resulted in only 33.4% mortality, which differed significantly from 83.4% mortality caused by CpGV-S and from the mortality observed in CpS with both viruses (ANOVA, Scheffé test, *p* < 0.05) (Table 2). This finding suggested that resistance of CpR5M is midgut-based for CpGV-S but systemic for CpGV-M.

### 3.4. ODV Infection Test and Fluorescence Dequenching Assay

**Figure 3:**
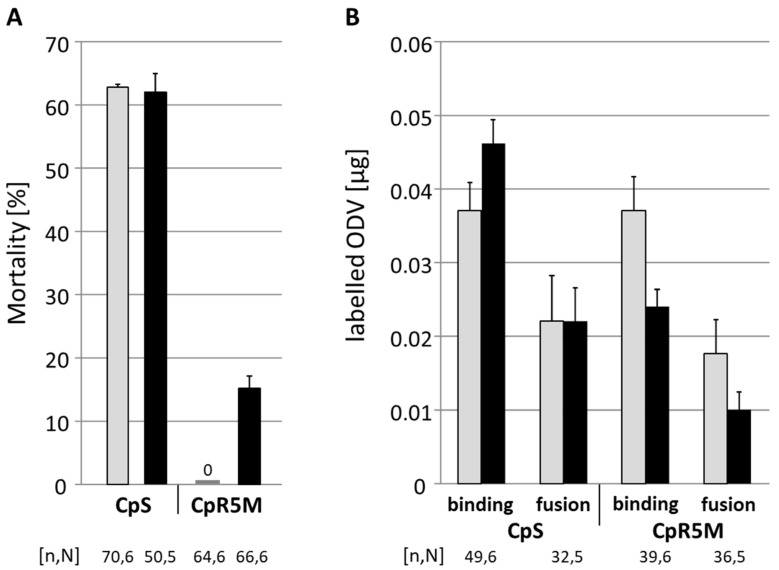
Mortality and results of fluorescence dequenching assays of CpS and CpR5M per os infected with labeled ODV. L4 larvae were orally infected with 2.4 µg of labeled ODV of CpGV-M (gray) or CpGV-S (black). (**A**) Abbott-corrected mean mortality and standard errors (error bars) were recorded 7 days post-infection. (**B**) Amount of labeled ODV bound or fused with midgut epithelial cells; dissection of midgut epithelial cells was undertaken 1 h post-infection. The total number of tested individuals (n) and number of independent replicates (N) are given below the chart.

To test whether the observed difference of midgut-related resistance to CpGV-M and CpGV-S depends on differences in the binding and fusion of ODV to the midgut epithelial cells in CpR5M, fluorescence dequenching assays were performed using R-18 labeled ODV prepared from CpGV-M and CpGV-S. This assay allows measuring of the RFUs associated with R-18 labeled ODVs fused to each midgut epithelial cell sample and its fusion capacity when treated with Triton-100. ODV preparations fed to L4 larvae of CpS caused mortality of 62% for both CpGV-M and CpGV-S, whereas in CpR5M larvae, only 0% and 15% mortality were recorded for CpGV-M and CpGV-S, respectively (Figure 3A). Thus, per os infection of larvae with the labeled ODV caused high mortality in CpS, but very low mortality in the resistant strain CpR5M. The fluorescence dequenching assay revealed for CpS larvae that an average of 0.046 µg ODV of CpGV-S and 0.037 µg ODV of CpGV-M bound to the midgut membrane; an average of 0.022 µg ODV of each virus fused with the midgut membrane (Figure 3B). In CpR5M, mean values for binding and fusion were lower for CpGV-S (0.024 µg in the binding and 0.010 µg in the fusion) than for CpGV-M (0.037 µg in binding and 0.017 µg in the fusion). Thus, binding and fusion of CpGV-M ODVs in CpS and CpR5M and binding and fusion of CpGV-S ODVs in CpS appeared similar, whereas an about 40% reduced ODV binding and fusion of CpGV-S was observed in CpR5M. However, these differences were not significant due to a high variation between the single measurements in the test replicates (Scheffé test, *p* < 0.05) (Figure 3B).

## 4. Discussion

In the present study, infection assays with OB, ODV, and BV of CpGV-M, CpGV-S alone, as well as of OB mixtures, were carried out to elucidate the mechanism of type II resistance in the codling moth strain CpR5M. Mixed infections of CpR5M with CpGV-M and CpGV-S did not result in a notable increase of mortality compared to single isolate infections. Thus, no synergistic interaction of CpGV-M and CpGV-S could be noticed in CpR5M. This finding is in contrast to the type I resistance, where some synergistic action and replication of CpGV-M (genome group A) and the resistance-breaking CpGV-R5 (genome group E) was noticed in co-infection experiments of larvae of the French type I-resistant colony RGV [30,42].

**Figure 4:**
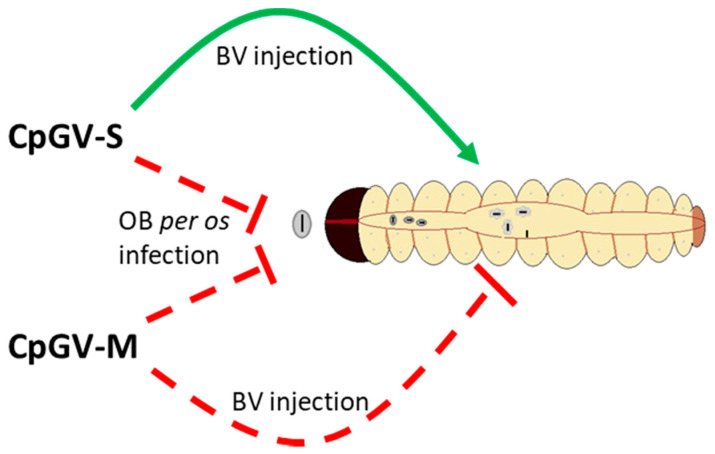
Schematic illustration of the hypothesis of two different resistance mechanisms in CpR5M larvae for resistance to CpGV-M and CpGV-S. Midgut infection of CpR5M with CpGV-M and CpGV-S is blocked as proposed by the peroral infection experiments (dashed red line) with OB (see Figure 1 and Figure 2). Systemic infection is blocked by CpGV-M, but not by CpGV-S, as indicated by budded virus injection (solid green line) (Table 2). If midgut is by-passed, CpGV-S is infective for CpR5M.

To investigate if there is a midgut factor of type II resistance, comparative BV injections into the hemocoel of CpS and CpR5M larvae were performed. Such BV injections are a very powerful method to discriminate midgut-based blocks of infection from systemic ones [33,43,44,45]. Indeed, injections of CpGV-S BV into CpR5M larvae caused high mortality, similar to that of BV injections of CpGV-S and CpGV-M into susceptible CpS larvae, clearly indicating that the midgut is an important barrier of CpGV-S infection in CpR5M. Mortality of CpR5M larvae injected with BV of CpGV-M was significantly lower. This different susceptibility of CpR5M to BV injections of CpGV-M and CpGV-S clearly indicates that resistance to CpGV-M and CpGV-S follows different mechanisms, a midgut-based mechanism for CpGV-S and a systemic mechanism for CpGV-M that cannot (or only at a reduced rate) be circumvented by BV injections (Figure 4). Previous BV injection experiments had demonstrated that type I resistance against CpGV-M is not midgut-based but is also systemic [33]. Thus, the BV infection experiment clearly supports the hypothesis of two different resistance mechanisms for CpGV-M and CpGV-S in CpR5M and that resistance against CpGV-S is indeed located in the midgut of CpR5M.

One conceivable explanation for a midgut-related resistance could be that the peritrophic membrane (PM), which acts as a physical barrier of the ODV passage from the gut lumen to the midgut epithelial cells [46], is changed in CpR5M. This possibility, however, would be very unlikely to explain midgut-based resistance against CpGV-S because it would require an isolate-dependent, selective sieving capacity of the PM. Midgut-based resistance could be the consequence of an isolate-specific disturbance of ODV attachment to midgut epithelial cells [40]. ODV binding and fusion is a highly complex process, which involves numerous baculovirus binding and fusion proteins as well as host receptors, which are so far not fully identified [47,48,49,50]. A mutation of a receptor molecule in the insect midgut resulting in an isolate-specific change of ODV binding may impair the ODV entry into the midgut cell and eventually impair larval susceptibility. Recently, a midgut-based resistance was demonstrated in laboratory-selected larvae of the tea tortrix *Adoxophyes honmai* to Adoxophyes honmai nucleopolyhedrovirus (AdhoNPV) by fluorescence dequenching assays [39,43,51]. However, when applying this assay with ODV derived from CpGV-M and CpGV-S, differences between the binding and fusion of CpGV-M and CpGV-S ODV in both CpR5M and CpS strains were statistically not significant. However, in CpR5M larvae, the mean binding and fusion capacity of ODVs from CpGV-S was about 40% lower than those of CpGV-M ODV. Whether this (statistically not confirmed) difference alone appears to be strong enough to explain the midgut-based resistance to CpGV-S needs to be quantified in further experiments. It is conceivable that another so far unknown blocking of virus infection is located in the midgut of CpR5M. This blocking may be intracellularly located in midgut epithelial cells, does not play a role in the BV driven systemic infection, and cannot be rescued in the midgut by co-infecting CpGV-M. The binding/fusion assays were a first step toward dissecting the resistance mechanism toward CpGV-M and -S in CpR5M. In any case, further investigations are necessary to elucidate the full picture of the midgut-based factor(s) of type II resistance.

The discovery of apparently two separate resistance mechanisms against CpGV-M and CpGV-S in CpR5M poses another question on the observed cross-resistance of CpR5M to both viruses [33]. If the two mechanisms are functionally not related, but were apparently co-selected and co-inherited as proposed by Sauer et al. [33], it is predicted that the underlying genetic factors must be located in close vicinity on the same autosome of the genome of CpR5M. In addition, the genetic factor(s) of resistance with pe38 as the target can be autosomally or Z-linked inherited, as observed for CpR5M and CpRR1, respectively. Whether this factor is genetically mobile or present at different chromosomal locations needs to be further investigated.

In summary, this study revealed a midgut-based mechanism of the novel, highly complex type II resistance of the codling moth against CpGV-S, but not to CpGV-M. Deciphering the molecular and cellular principles of baculovirus resistance, and how these factors are established and selected in the codling moth field populations, will be a great asset to better understand baculovirus-insect interaction on organismic and population level.

## Figures and Tables

**Table 1:** Mortality of L4 Larvae of CpS Injected with Budded Virus of CpGV-M after 14 Days.

Concentration (BV/Larvae)	Number of CpS Larvae	% Mortality
50	14	19.6
500	15	58.3
5000	19	73.6
50,000	17	92.7
500,000	14	100.0

**Table 2:** Mortality of L4 Larvae of CpS and CpR5M after Budded virus (BV) injection of CpGV-M and CpGV-S. Larvae were injected with 5 × 10^3^ BV/larvae into the hemocoel. Given is the Abbott-corrected mean mortality at 14 days post injection (p.i.), standard deviation (±SD), number of tested individuals (n), and number of independent replicates (N). Different letters indicate statistical differences in the means following ANOVA, Scheffé test (*p* < 0.05).

Codling Moth Strain	BV Treatment	n, N	% Mortality * (±SD) 14 Days p.i.	Test for Significant Statistical Differences
CpR5M	CpGV-M	61, 4	33.4 (±6.4)	A
	CpGV-S	42, 3	83.4 (±7.5)	B
CpS	CpGV-M	57, 4	73.2 (±10.9)	B
	CpGV-S	41, 3	(±5.9)	B

* Mortality in the control group with injection of uninfected hemolymph from CpS was 31.1% for CpS and 8.0% for CpR5M.

## Data Availability

The data presented in this study are available on request from the corresponding author.
